# Cow’s Milk: A Benefit for Human Health? Omics Tools and Precision Nutrition for Lactose Intolerance Management

**DOI:** 10.3390/nu16020320

**Published:** 2024-01-22

**Authors:** Giovanni Pratelli, Bartolo Tamburini, Giusto Davide Badami, Marianna Lo Pizzo, Anna De Blasio, Daniela Carlisi, Diana Di Liberto

**Affiliations:** 1 Department of Biomedicine, Neurosciences and Advanced Diagnostics (BIND), Institute of Biochemistry, University of Palermo, 90127 Palermo, Italy; giovanni.pratelli@unipa.it (G.P.); daniela.carlisi@unipa.it (D.C.); 2 Department of Health Promotion, Mother and Child Care, Internal Medicine and Medical Specialties (ProMISE), University of Palermo, 90127 Palermo, Italy; bartolo.tamburini@unipa.it; 3 Central Laboratory of Advanced Diagnosis and Biomedical Research (CLADIBIOR), AOUP Paolo Giaccone, 90127 Palermo, Italy; giustodavide.badami@unipa.it (G.D.B.); marianna.lopizzo@unipa.it (M.L.P.); 4 Department of Biological, Chemical and Pharmaceutical Sciences and Technologies (STEBICEF), Laboratory of Biochemistry, University of Palermo, 90127 Palermo, Italy; anna.deblasio@unipa.it

**Keywords:** cow’s milk, lactose intolerance, microbiota, food allergy, inflammation, omics

## Abstract

Cow’s milk (CM) is a healthy food consumed worldwide by individuals of all ages. Unfortunately, “lactase-deficient” individuals cannot digest milk’s main carbohydrate, lactose, depriving themselves of highly beneficial milk proteins like casein, lactoalbumin, and lactoglobulin due to lactose intolerance (LI), while other individuals develop allergies specifically against these proteins (CMPA). The management of these conditions differs, and an inappropriate diagnosis or treatment may have significant implications for the patients, especially if they are infants or very young children, resulting in unnecessary dietary restrictions or avoidable adverse reactions. Omics technologies play a pivotal role in elucidating the intricate interactions between nutrients and the human body, spanning from genetic factors to the microbiota profile and metabolites. This comprehensive approach enables the precise delineation and identification of distinct cohorts of individuals with specific dietary requirements, so that tailored nutrition strategies can be developed. This is what is called personalized nutrition or precision nutrition (PN), the area of nutrition that focuses on the effects of nutrients on the genome, proteome, and metabolome, promoting well-being and health, preventing diseases, reducing chronic disease incidence, and increasing life expectancy. Here, we report the opinion of the scientific community proposing to replace the “one size fits all” approach with tailor-made nutrition programs, designed by integrating nutrigenomic data together with clinical parameters and microbiota profiles, taking into account the individual lactose tolerance threshold and needs in terms of specific nutrients intake. This customized approach could help LI patients to improve their quality of life, overcoming depression or anxiety often resulting from the individual perception of this condition as different from a normal state.

## 1. What Is Cow’s Milk Made of?

Cow’s milk (CM) is our first food, although milk and its numerous derivatives are still consumed in adulthood and old age. However, whether milk is a healthy or unhealthy food compared to others is constantly being debated in the scientific community, and its consumption is often the subject of controversy.

**Figure 1:**
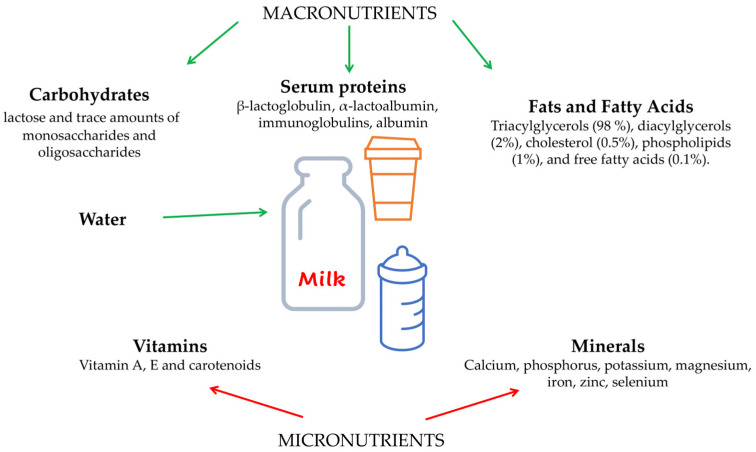
Schematic representation showing the components of cow milk. Parts of the figure were drawn using pictures from Server Medical Art. Servier Medical Art by Servier is licensed under a Creative Commons Attribution 3.0 Unported License.

It is true that humans are the only adult mammals who consume milk after weaning, but it is undeniable that it is a complete food from a nutritional perspective due to its high amounts of macronutrients, such as proteins, lipids and carbohydrates, and micronutrients, such as vitamins and minerals [1] (Figure 1). An extensive network of production and distribution ensures that milk is available to the consumer in all forms, fresh or long-life, depending on the method of processing. According to the “Codex Alimentarius Commission” instituted by the Food and Agriculture Organization of the United Nations (FAO) and the World Health Organization (WHO), “Milk is the normal mammary secretion of milking animals obtained from one or more milkings without either addition to it or extraction from it intended for consumption as liquid milk or for further processing”. (https://www.fao.org accessed on 11 December 2023).

However, there are different types of drinking milk commercially available that meet the needs of all consumers. There are, in fact, several types of milk modified in composition by the addition and/or withdrawal of some milk constituents, such as low-calorie milk, low-protein infant formula and obesity-risk milk, completely skimmed milk or milk supplemented with omega 3 fatty acids (FAs), calcium and protein. Therefore, milk products derive from different types of milk processing and may contain food additives and other functional ingredients necessary for the process. The aims of these adjustments, in terms of addition or withdrawal, are to protect the consumer and ensure the correct use of milk and milk products at all ages. It has been reported that protein intake during the first 6 months of life is higher in formula-fed infants (approximately 70%) when compared to breastfed infants [2].

It is thought that the lower protein concentration of breast milk can positively influence infant growth, possibly preventing childhood obesity- and adulthood obesity-related disorders. Currently, research is focused on understanding the underlying mechanisms of early protein intake on later health and the most appropriate infant formula for improved protein quality together with a reduced protein content for optimal growth. However, breast milk remains the gold standard of infant nutrition, providing a unique combination of nutrients and more. CM is considered by some to be the perfect food, not only for its nutritional value, but also for its hydration properties and its contribution to maintaining the stability of the intestinal microbiota and priming the immune system in infants [3].

CM and its derived dairy products play a significant role in providing a high-nutrient food source, with a positive impact on human health. The human diet typically includes milk as a source of proteins and microelements such as calcium. The composition of bovine milk can vary significantly in relation to many variables, such as cattle breed, lactation period, parity, and the animal’s health status [4]. Several environmental factors, such as nutrition type, can play a major role in the nutritional and organoleptic qualities of milk, both as a drink and as a foundation for dairy products. The chemical composition of milk includes a water content of 80% or more, as well as proteins, lipids, sugar, minerals, and vitamins (Figure 1).

Foroutan et al. performed a complete and exhaustive analysis on the chemical composition of commercial CM, taking into account the assessment of metabolites and macromolecules. In summary, the compositional analysis of milk reveals a set of key constituents, such as carbohydrates (lactose, glucose, and galactose), inorganic ions (potassium and calcium), organic acids (citrate) and amine-containing compounds (creatinine, choline, and urea). Smaller quantities of vitamins, triacylglycerols (TGAs) the dominant lipid components, di- and monoacylglycerols, FAs, short-chain fatty acids (SCFAs), amino acids, and other small bioactive compounds are also evident. In addition, milk also contains a number of macromolecules, including DNA and RNA, and several proteins such as the bovine casein peptides, β-lactoglobulin, and α-lactalbumin [5].

## 2. Cow’s Milk Proteins

The milk proteins have a high nutritional value, being rich in essentials amino acids (leucine, isoleucine, valine, lysine, histidine, methionine, and phenylalanine) [1]. Milk proteins, separated using membrane microfiltration procedures, may be found in the soluble whey fraction (20%), and in the insoluble micellar caseins fraction (80%). Whey proteins, also called serum proteins, include β-lactoglobulin (β-LG), α-lactoalbumin (α-LA), immunoglobulins (IgG1, IgG2, IgA, and IgM), serum albumin (BSA), and to a lesser extent, β-lactoferrin (β-LF), lactoperoxidase, lysozyme, vitamin-binding and metal-binding proteins, and hormones. They all possess a wide variety of biological significant functions [6,7], acting as health promoters. In particular, β-LG works as an immunoglobulin carrier during colostrum formation, α-LA may act as a lactose synthase component also having antimicrobial and anticancer activity, β-LF is a glycoprotein with iron binding and delivery functions transferrin-like and, moreover, exerting antimicrobial, antiviral, immunomodulatory, antioxidant and antitumor activity, lysozyme has antimicrobial functions, immunoglobulins protect the mammary gland from infections, BSA has anticancer and immunomodulatory activity and, finally, lactoperoxidase has antimicrobial and antioxidant properties. In the soluble fraction containing whey proteins, lactose, salts, vitamins, and trace amounts of other compounds are also found [8,9].

The insoluble micellar fraction of milk proteins is constituted by caseins that are present in milk in the form of self-assembled colloidal particles of 50–600 nm in diameter, known as “casein micelles”, and distinguished as αs1-casein, αs2-casein, β-casein, γ-casein, and κ-casein [10]. The micellar protein fraction has many bioactive functions and is responsible for several characteristics of milk, such as white color or coagulation capacity or heath resistance. Casein micelles are structurally complex and are able to carry calcium and phosphate, with κ-casein located on the globule surface, determining the size of the micelle, as well as preventing their aggregation [11].

β-casein is encoded by the CNS2 gene and 13 different allelic variants (A1, A2, A3, B, C, D, E, F, G, H1, H2, and J) have been described. A1 and A2 β-casein are the two most common variants, differing by only one amino acid at position 67. The A1 variant is only present in cattle, while the A2 variant is present in the milk of many mammals, including humans. Dairy cow breeds present different β-casein patterns in their milk and, nowadays, most milk marketed contains a mixture of A1 and A2 β-casein.

A2 milk appears to be healthier than A1 due to its natural antioxidant activity, resulting in glutathione production increase [12], and its lower amount of bioactive opioid peptide β-casomorphin 7 (BCM-7) released upon digestion, compared to A1 milk. BCM-7 is harmful for human health, due to its opioid-like activity as a result of binding to µ-opioid receptors, being responsible for pain and symptoms in the gastrointestinal tract as well as in the nervous system.

In addition, as reported in mice models, β-casein A1, compared to the β-casein A2, can increase the inflammatory response as well as intestinal permeability and IL-4 production by activation of the Th2 pathway [13]. Moreover, by measuring the TNF-α and histamine release from Human Mast Cells (HMC)-1 cells, the hypoallergenic property of A2 β-casein was demonstrated [14].

In fact, it was reported that α-caseins, β-caseins, and β-lactoglobulin inhibit cellular senescence, playing a protective role against oxidative damage of cellular components that results from the accumulation of Radical Oxygen Species (ROS), namely oxidative stress, thus preventing aging-associated diseases, or skeletal muscle loss. In addition, the enzymatic hydrolysis of several proteins generates many bioactive peptides that can exert protective functions, enhancing human well-being and health [15].

## 3. Cow’s Milk Fats and Micronutrients

CM fats are mainly constituted by TAGs (98%), diacylglycerols (DAGs) (2%), cholesterol (0.5%), phospholipids (1%), and free FAs (0.1%). The wide presence of phospholipids in milk provides numerous benefits to human health, due to their structural and functional role in the cell membrane and in cell signaling, respectively. In milk, there are more than 30 classes of phospholipids, such as phosphatidylcholine, phosphatidylethanolamine, phosphatidylserine, phosphatidylinositol, and sphingophospholipid. In particular, these molecules are found within two fractions of milk: in the trilayer membrane surrounding fat globules and in nanovesicles secreted into milk by cells of the mammary gland [16]. To date, special attention has been given to milk-derived exosomes (MDEs), phospholipid bilayer nanovesicles released from breast milk. MDEs have been found in humans, cows, pigs, and rats and they seem to be involved in multiple biological processes. They are present at different lactation periods, such as colostrum or mature milk, and contain proteins, lipids, DNA, mRNA, microRNA and long non-coding RNA (lncRNA), thus protecting them against enzymatic and non-enzymatic degradation. MDEs play an emerging role as messengers between cells and, although little information is available about the role of lncRNA in MDEs, they are involved in gene expression and development [16]. Feng et al. reported that MDEs play an important role in the development and immune functions of the digestive tract thanks to their capacity to transfer their cargos to the cytoplasm of cell targets by endocytosis. Moreover, they report the hypothesis of their application as nanodevices for the development of new chemotherapeutic/chemopreventive carriers [17].

Sphingophospholipid is the major fat type present in milk [18], affecting human health in many different ways. In particular, a high concentration of sphingomyelin present in the phospholipid fraction of milk is important for neuronal development and protection from bacterial infections of neonates. In this fraction, there are also gangliosides, another kind of sphingolipid, that have potential bioactivities in brain function and the immune system [19,20].

The high complexity of fat milk derives from the wide variety of FAs bound to either the glycerol or sphingosine backbone. There are many variables that can be connected with FA amount and the composition of bovine milk, because they can be derived from either food or rumen microbiota activity and influenced by animal origin, stage of lactation, seasons and mastitis. Generally, the composition of the fat fraction is: 70% saturated (palmitic, myristic, stearic and SCFAs) and 30% unsaturated FAs (oleic, linoleic, a-linolenic) and a certain amount of trans-fatty acids (vaccenic acid).

Carbon chains length of FAs can vary from C2 to C24 and they can be saturated (SFA), monosaturated (MUFA) or poly-unsaturated (PUFA) due to the presence of single, for saturated, or at least one or more double bonds between the carbons for MUFA and PUFA, respectively. PUFA, like omega-3s, with the last double bond at the third carbon from the omega end of the chain, and omega-6s, with the double bond at the sixth carbon from the omega end of the chain, are considered “good” fats and an important part of a healthy diet because they are “essential”, since humans, and other animals, cannot synthesize them and must introduce them with the diet. The “optimal” ratio of omega-6s to omega-3s, as proposed by Benbrook et al., is suggested to be approximately 2:1 [21]. This balance is crucial, as an excess of omega-6s can prevent the effective utilization of omega-3s, consequently restricting their manifold health advantages. These benefits include mitigating the risk of cardiovascular disease, diabetes, and obesity. Although milk can be fortified with plant-based omega-3s, researchers are currently investigating the potential benefits of fortifying dairy cattle diets with omega-3s to influence the proportion of these healthy fats in milk.

The question arises whether an increased intake of omega-3s by cows corresponds to an elevation of omega-3s in their milk. While this relationship is true in humans and mammals, the dynamics between diet and milk composition in cows are complex and require further exploration [22]. As cows digest the fats in their food, the microbial activity in their rumen converts the double bonds of PUFA into single bonds, turning them into saturated FAs [21]. To this end, researchers are currently experimenting with different cow feeding with different combinations and concentrations of various vegetable sources of omega-3s and the results are promising.

Finally, in CM, there are also traces amounts of hydrocarbons, fat-soluble vitamins, flavor compounds, and a wide range of micronutrients, including minerals, such as calcium, phosphorus, potassium, magnesium, iron, zinc, selenium, as well as vitamins, such as vitamin A, E and carotenoids [23]. Some of them, like selenium, zinc, vitamin E, C, beta-carotene and sulfur, exert an important antioxidant activity themselves or as cofactors of antioxidant enzymes, like glutathione peroxidase, superoxide dismutase and catalase.

Therefore, several milk constituents have an antioxidant activity useful for preventing the consumer from metabolic and chronic ROS-related diseases such as diabetes, atherosclerosis and cancer.

Nowadays, the comprehensive association between dietary intake and diseases is not only complex but is also not easy to understand, with controversial scientific evidence. For example, although milk consumption is recommended for its high calcium content resulting in an increase in bone density and thus preventing the onset of osteoporosis, currently there is also considerable debate concerning the effect of calcium supplementation by itself in bone mass [24].

Recent studies have linked calcium supplements with an increased risk of colon polyps, kidney stones and cardiovascular disease (CVD) risk [25,26]; on the other hand, a meta-analysis of all placebo-controlled randomized trials demonstrated that there is not a significant association between calcium supplements, alone or with vitamin D, and risk for CVD and all-cause mortality [27].

In addition, in meta-analysis studies, Drouin-Chartier et al. report neutral associations between the consumption of various forms of dairy products and CVD, as well as favorable associations between total dairy intake and hypertension risk [28].

Furthermore, some studies reported that milk consumption could increase the risk of several Western diseases, including metabolic diseases such as diabetes [29,30], while other studies show that dairy consumption was inversely associated with the risk of developing cancer, including breast and colorectal cancer [31,32].

Nonetheless, it is unquestionable that, due to its chemical and biochemical composition, CM and its derivatives are a source of important macro- and micronutrients, suggesting that its consumption is crucial for development during the first years of life and still relevant in adulthood [33].

## 4. IgE-Mediated Immune Response to Cow’s Milk Proteins

**Figure 2:**
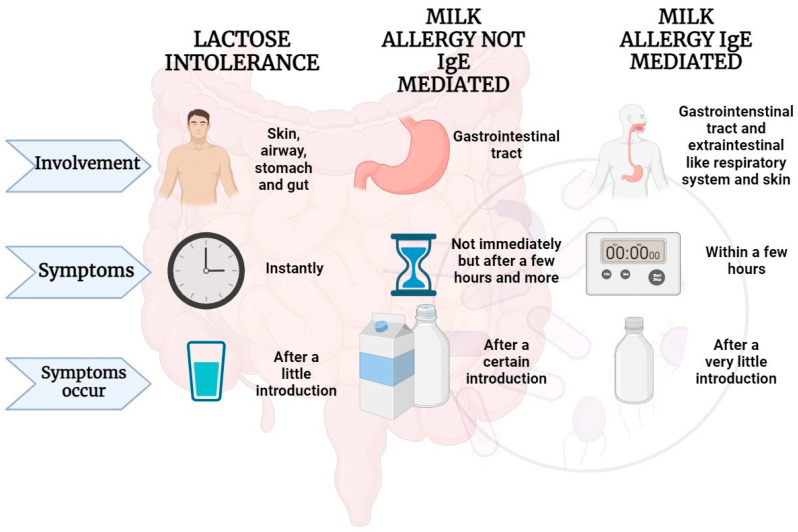
Adverse effects that may occur following milk intake in some predisposed individuals. Parts of the figure were drawn by using BioRender.com.

As a pivotal element in the human diet, CM stands as a significant factor contributing to numerous gastrointestinal disorders [34]. Several constituents found in CM, including lactose and bovine milk proteins, have been identified as potential triggers for gastrointestinal discomfort and disorders. Among the prevalent conditions associated with milk intake are cow’s milk protein allergy (CMPA) and lactose intolerance (LI) (Figure 2).

CMPA is the predominant food allergy observed during infancy and early childhood, exhibiting an estimated prevalence among children under three years ranging from 0.5% to 3% in developed nations [35,36]. In general, CMPA typically manifests within the initial year of life, showing an average onset age of 3.9 months and exhibiting a promising prognosis, as the majority of affected children tend to develop tolerance by the age of four [37,38,39]. Nevertheless, within a subset of patients, CMPA persists into adolescence and adulthood, leading to severe allergic responses even upon exposure to minute traces of milk [40].

CMPA represents an immunopathogenic reaction elicited by milk proteins. Following initial exposure to specific milk proteins, the immune system identifies them as allergens, subsequently prompting the manifestation of CMPA signs and symptoms. CMPA is categorized into three distinct types of contingent upon the nature of the immune response: (i) Immunoglobulin E (IgE)-mediated, with IgE antibodies directed against milk proteins. (ii) Non-IgE (or cell-mediated), wherein the allergic reaction is orchestrated by the cellular immune system, particularly T cells. (iii) A mixed-type reaction, characterized by the involvement of both IgE antibodies and immune cells in the allergic response [41].

CM consists of an array of over 20 protein fractions. The predominant allergens are caseins (α1-, α2-, β, and κ-casein), constituting 20% of the total protein, and whey proteins. Many individuals sensitive to CM proteins exhibit reactivity to both. Each protein possesses the potential to act as an allergen, provoking both IgE- and non-IgE-mediated immune responses, consequently triggering a diverse array of clinical manifestations and distinctive phenotypes [42].

The predominant form of CMPA is the IgE-mediated variant, constituting approximately 60% of all allergic reactions induced by CM [43]. This allergic reaction is initiated by the generation of specific IgE antibodies targeting CM proteins, which subsequently bind to high-affinity IgE receptors (FεRI) found on basophils and mast cells. Upon contact with CM proteins, two or more specific IgE antibodies bind to FεRI, leading to receptor cross-linking and subsequent activation of mast cells in the skin, gut, respiratory, and cardiovascular systems. This activation prompts the release of mediators like histamine, leukotrienes, and cytokines, which orchestrate an inflammatory cascade.

IgE-mediated responses commonly manifest immediately following CM ingestion or within a span of 1 to 2 h. The mediators initiate vasodilation, triggering acute manifestations; the skin may exhibit localized reactions like hives or rashes, while the gastrointestinal tract might manifest symptoms like nausea, vomiting, or diarrhea. Respiratory responses can include coughing, wheezing, or shortness of breath, while the cardiovascular system may experience changes in blood pressure or heart rate.

This reactivity, known as an allergic reaction, is an adaptive defense mechanism of the body but can result in discomfort or, in severe cases, life-threatening conditions such as anaphylaxis [41,44]. The diagnosis of IgE-mediated CMPA relies on a comprehensive assessment, combining a history of indicative symptoms, physical examinations, and the identification of CM sensitization through the detection of specific CM proteins IgE. This determination is achieved via skin prick tests to observe the presence of CM-specific IgE in mast cells within the skin or through serum analysis [45].

Differently from IgE-mediated CMPA, the non-IgE-mediated immune response impacts various segments of the gastrointestinal tract (GT) and is characterized by symptoms that manifest with a delayed onset, occurring anywhere from 2 h to several days following CM ingestion. Clinical manifestations of this response include allergic proctocolitis, induced enterocolitis syndrome, and induced enteropathy.

**Figure 3:**
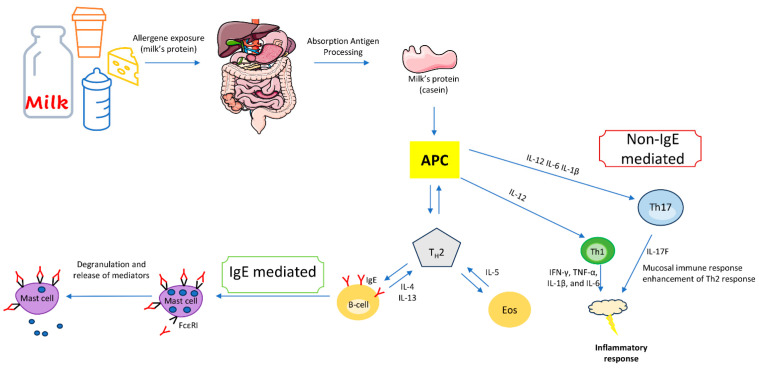
Model for the onset of IgE- and non-IgE-mediated cow’s milk protein allergy. Parts of the figure were drawn using pictures from Server Medical Art. Servier Medical Art by Servier is licensed under a Creative Commons Attribution 3.0 Unported License.

Mixed types of food allergies are characterized by the involvement of both IgE antibodies and various immune cells including eosinophils, T lymphocytes, and dendritic cells resulting in a diverse range of symptoms affecting multiple organ systems. These cells further exacerbate the immune reaction by releasing additional inflammatory factors, amplifying the overall immune response, and potentially causing prolonged or more severe allergic manifestations. Particularly food allergy driven by Th2 responses, marked by elevated levels of IL-5, IL-13, and IL-9 (Figure 3); this condition exhibits increased numbers of eosinophils, mucosal mast cells, and CD4^+^ T cells within the specific tissue [46].

This kind of reaction encompasses a cluster of conditions commonly categorized under eosinophilic gastrointestinal disorders (EGID), which include ailments like eosinophilic esophagitis, gastritis, gastroenteritis, and colitis [47].

The onset of all food allergies is shaped by a combination of genetic factors, environmental elements, and genome–environment interplays, incorporating significant epigenetic influences. Certain unmodifiable risk factors heighten the susceptibility to developing food allergies, including gender (male predominance in children), racial or ethnic background (higher prevalence observed among Asian and black children in comparison to white children), and familial history of atopic conditions [48]. Additional potential risk factors warranting examination for the reduction or prevention of food allergies include heightened hygiene practices, the impact of the microbiota [49], dietary fat, diminished consumption of antioxidants, heightened utilization of antacids (impacting allergen digestion), obesity (associated with an inflammatory state), and the timing of food introduction in the diet. Delayed oral exposure to allergens in the absence of concomitant environmental exposure may lead to sensitization and subsequent development of allergies. For this reason, a progressive introduction of foods diversity is recommended during weaning. Indeed, several studies have indicated that a protective element against sensitization and the consequent allergic response lies in the diversification of foods introduced during early childhood [50].

The introduction of multiple allergenic foods by the age of 6 months seems feasible, as several studies have indicated [51]. Children exposed to a diverse range of foods and food allergens during their first year of life might experience a reduced risk of developing food allergies throughout the initial decade of life [52].

It is estimated that the risk of developing a food allergy escalates to 40% in individuals with a single immediate family member affected by any allergic disease. In cases where two immediate family members are impacted by allergic conditions, this risk spikes to 80%, contrasting significantly with children lacking a family history of allergies [53]. As observed for all food allergies, it is widely acknowledged that atopic conditions, especially atopic dermatitis, constitute a significant risk factor for IgE-mediated CMPA [54].

Comorbid atopic conditions like asthma, particularly when poorly managed, correlate with frequent and severe reactions to milk [55].

## 5. Non-IgE-Mediated Immune Response to Milk Proteins

CMPA can also manifest through non-IgE-mediated immune responses, which are different from the typical IgE-mediated allergic reactions seen in immediate hypersensitivity reactions [56] (Figure 3). Non-IgE-mediated CM allergies often involve delayed and chronic symptoms and are less common than IgE-mediated allergies and the immunological mechanisms involved are not well understood. In non-IgE-mediated CMPA, T cells play a significant role.

When CM proteins are ingested, T cells can react against them, triggering inflammation and immune responses. This response is delayed and can lead to various symptoms, including gastrointestinal issues, such as chronic diarrhea, abdominal pain, and malabsorption of nutrients [57]. Limited research has demonstrated the generation of Th1 polarization cytokines (including IFN-γ, TNF-α, IL-1β, and IL-6) in CMPA [58]. The transition of CD4^+^ lymphocytes towards a Th1 response, triggered by IL-12, might also contribute to the pathogenesis [59]. Several studies propose that beyond the Th2-mediated immune responses involved in the production of IL-4, IL-5, and IL-13, there may be additional mechanisms at play. One such mechanism involves alterations in intestinal motility, believed to arise from the intricate interplay between lymphocytes, mastocytes, and the enteric nervous system [56,57,58].

Previous studies have indicated elevated IFN-γ production in duodenal biopsies among children afflicted with both food allergies and CMPA [60,61] and recent studies revealed heightened levels of IL-6 and CCR4 along with reduced levels of IL-18 and IL-2 in duodenal biopsies from children experiencing non-IgE-mediated CMPA [62].

Th17 polarization has yet to be observed in the context of food allergy. Among the central cytokines produced by Th17 subpopulations, IL-17 stands out. This cytokine family consists of six distinct subtypes, ranging from IL-17A to IL-17F. Although IL-17A and IL-17F share a significant portion of their amino acids, their functions diverge. While IL-17A is implicated in inflammatory processes such as autoimmunity, cancer immunity, and defense against bacterial and fungal infections, IL-17F is specifically associated with mucosal immunity and amplifying the Th2 response. Given these distinctions, it can be considered essential to investigate the response of IL-17F in patients with CMPA [63].

In another recent study, researchers examined the differential gene expression of Th1, Th2, and Th17 cytokines in the duodenum and rectum of patients under 2 years old with non-IgE-mediated CMPA. Taken together, all these findings suggest that the immune response in these patients is influenced by this specific cytokine profile, with the rectum identified as the primary affected site [57].

In addition, CMPA can also involve IgG-mediated immune responses, which are different from the classic IgE-mediated allergies and non-IgE-mediated immune responses. Also, IgG-mediated immune responses are generally associated with delayed or non-immediate allergic reactions, and their role in CMPA is a subject of ongoing research.

Recently, two research groups have documented the existence of specific IgG4 (sIgG4) antibodies targeting food extracts [64,65], although sIgG4 possess structural attributes that facilitate their anti-inflammatory activity, positioning them as mediators associated with the tolerance of allergens [66]. Despite these findings, the clinical implications remain unclear. This ambiguity is partly attributed to the insufficient understanding of the prevalence in the general population of sIgG4 antibodies against food proteins combined with a lack of a concurrent analysis of IgE antibodies, which are traditionally associated to immediate allergic reactions.

## 6. Microbiota and Lactose Intolerance

Nutrients introduced with food interact with the human body in the GT, by their digestion and absorption [67]. GT is a sort of physical barrier against many potentially harmful ingested substances whose integrity is crucially involved in the health and defense of the human body. Nowadays, it is well known that everything that in any way alters or disrupts microbiota composition, such as molecules from foods, chemical substances, or other types of stressors, has the potential to change its functionality, harming people’s health [68]. However, dietary nutrients and their interactions with bacteria colonizing GT are responsible for health benefit for the host, named “symbiosis”, such as the ability to metabolize lactose and other carbohydrates in the small intestine to generate glucose as energy source. Otherwise, alterations in the composition and functions of gut microbiota, named “dysbiosis”, may lead to the insurgence of chronic metabolic or immune-related disorders, and to several nutrients intolerance, such as LI [69].

GT is inhabited by a diverse array of microorganisms, primarily bacteria, which play crucial roles in metabolic, immunological, and gut-protective functions. The composition of these intricate microbial communities exhibits variation along the GT, with the colon showcasing heightened diversity, reaching a bacterial density of 10^12^–10^14^ colony-forming units (CFU)/mL. The intestinal microbiota is established at birth and undergoes dynamic changes, particularly during the initial years of life. In neonates, it is initially dominated by Proteobacteria, notably Escherichia, and Actinobacteria, including in particular *Bifidobacterium*. The composition of the microbiota undergoes continuous fluctuations and increases in diversity throughout childhood, achieving significant maturity by the age of two to three years. Following this phase, it tends to adopt a more stable composition, with Firmicutes and Bacteroidetes emerging as dominant phyla, each characterized by distinct functions [70].

Despite this diversity, the human gut predominantly comprises six major phyla: Firmicutes, Bacteroidetes, Actinobacteria, Proteobacteria, Tenericutes and Fusobacteria [71,72,73]. However, these values exhibit variations in the infant intestine, where Actinobacteria, particularly the genus *Bifidobacterium*, typically dominate. Furthermore, the adult gut microbiota is characterized by greater complexity in terms of both total bacterial abundance and the encountered diversity of microbial taxa. A comparable pattern is noted in the gut microbiota of the elderly population, where microbial intestinal communities seem to display reduced diversity [73]. Nonetheless, the precise taxonomic makeup is contingent on various host-related factors, including genetic variation, age, diet, and geographic location, leading to significant variability among healthy individuals [74]. In particular, bacterial communities exhibit both quantitative and qualitative variations influenced by host-related chemical-physical factors (i.e., bile acids, pH, transit time and mucus), environmental factors (drugs and foods), and microbial factors (i.e., bacterial enzymes, adhesion capability and metabolic strategies) [73].

Thus, lactose assumes a pivotal role in the intestinal microbiota from the early years of life as a structural component of human milk oligosaccharides (HMO). Each HMO molecule contains a lactose core, subject to cleavage by Bifidobacteria, including *B. brevis*, *B. infantis*, *B. longum*, and *B. lactis*, facilitated by the enzyme β-hexosaminidase. Then, β-galactosidase comes into play, hydrolyzing lactose into glucose and galactose [75]. The intestinal microbiota thus holds a central position in carbohydrate breakdown, particularly for complex vegetable carbohydrates that evade host digestion. Simultaneously, it contributes significantly to the proper development of the immune system, acting as a defense against pathogens.

Literature evidence supports the notion that the intestinal microbiota induces the production of IgA, thereby preserving the homeostasis of various populations of intestinal immune cells, including regulatory T cells (Tregs), a T cell subset specialized in suppressing immune response and maintaining self-tolerance and tissue homeostasis, helper T cells, and MAIT cells [73,76,77]. Indeed, the composition of nutrients intricately interacts with the immune defenses of the intestinal mucosa and both inflammatory and non-inflammatory cells, shaping their responses. For instance, the fibers and starches present in vegetables and fruits serve as substrates for the production of SCFAs by intestinal microbes. Among these SCFAs, butyrate, a by-product of dietary fiber digestion by the intestinal microbiota, but also produced by lactose-fermenting colonic commensal bacteria, assumes a pivotal role in the immune system. It stimulates the production of Tregs in the lamina propria, enhancing the barrier function of gut epithelium, while inhibiting the transcription of inflammatory cytokines [78,79].

Not only is butyrate a preferred energy source for enterocytes, but it also holds significant importance in maintaining epithelial homeostasis. Diminished levels of butyrate and dietary fiber, in general, expedite catabolism at the mucosal level, leading to heightened intestinal permeability. This increased permeability renders the mucosa more susceptible to potential luminal pathogenic bacteria [80,81].

As reported by Zhong and colleagues, an increased number of colonic bacteria with a higher lactose-fermenting ability seems to be related to a reduction in LI symptoms, as diarrhea occurrence [82]. This finding explains why some prebiotics, such as galacto-oligosaccharides (GOS), a substrate selectively employed by host microorganisms to impart a health benefit, ameliorate LI symptoms, being fermented by specific species of commensal colonic bacteria, such as *Lactobacillus* and *Bifidobacterium* [83,84]. It has been reported that GOS stimulate the growth and the activity of colonic bacteria that metabolize lactose by increasing its fermentation into glucose, galactose and SCFAs [84]. This process, called “colonic adaptation”, improves lactose digestion and tolerance, leading to a consequent reduction in gas production in the large intestine [85].

Differently, probiotics are live microorganisms, usually yeasts and bacteria, with the ability to promote a correct balance between the species that colonize our intestine and to guarantee health benefits. Probiotics are therefore useful for improving the digestion of nutrients and strengthening the immune system, and for this reason they are considered a valid therapeutic strategy [86]. Some of them, such as *Lactobacillus acidophilus* that we introduce by eating yogurt, own a beta-galactosidase hydrolytic activity able to relieve LI symptoms enhancing lactose metabolism [87] and the effectiveness is increased by the combination of several strains owning this hydrolytic activity [88]. It would therefore be ideal to design therapeutic LI strategies that make use of both probiotics and prebiotics selected to improve lactose digestion in order to alleviate LI symptoms [89].

Notably, there exists substantial variability in the severity of LI clinical manifestations both within and between individuals. It is crucial to recognize that these gastrointestinal symptoms may resemble those of CMPA, potentially leading to mislabeling as “milk allergy”. However, it is imperative to distinguish between LI and CMPA, as they represent distinct conditions. Enhancing the comprehension of these disparities is vital to prevent misunderstandings in both the diagnostic process and the management of these conditions [90].

Differently from LI, CMPA can produce different effects on the intestinal microbiota. In particular, many researchers have shown that individuals with CMPA may have differences in the composition of their intestinal microbiota compared to those without allergy. These differences can include alterations in the abundance of certain bacterial species or changes in overall microbial diversity [91]. Furthermore, several studies propose that individuals affected by CMPA may exhibit a lower grade diversity of gut microbiota. A decrease in microbial diversity has been linked to various health issues and could contribute to the onset of intestinal inflammation and allergies. Additionally, the gut microbiota plays a pivotal role in instructing and adjusting the immune system. Alterations in the microbiota composition in individuals with CMPA may impact the immune response, potentially leading to allergic reactions [47,91].

Finally, the gut microbiota is crucial for maintaining the integrity of the intestinal barrier. CMPA can result in intestinal inflammation, which may compromise the function of the gut barrier. A compromised gut barrier facilitates the easier passage of allergens, thereby intensifying the allergic response. Notably, certain beneficial bacteria, such as *Lactobacillus* and *Bifidobacterium* species, are prevalent in the fecal microbiota of healthy breastfed infants. In contrast, individuals with CMPA exhibit gut dysbiosis and an increased likelihood of developing other allergic conditions during later childhood. Although the hypothesis regarding causality requires further assessment, it is plausible that early gut dysbiosis disrupts regulatory mechanisms in the immune response, triggering pro-allergic processes and elevating the risk of allergies [92,93].

In the pursuit of precise and personalized medicine or precision nutrition (PN), investigating the modification of the gut microbiota emerges as a potential strategy in managing CMPA [91]. Dietary restrictions stemming from CMPA can exert a profound influence on the composition of the gut microbiota. Alterations in the diet, particularly the exclusion of CM and dairy products, have the potential to reshape the types of bacteria thriving in the gastrointestinal tract. Notably, this also includes the use of probiotics, and prebiotics as potential strategy helping us to rejuvenate a more robust intestinal microbiota. The objective is to foster a microbial environment conducive to overall well-being, specifically in addressing the complexities associated with CMPA [94].

## 7. Lactase Deficiency and Lactose Intolerance

**Figure 4:**
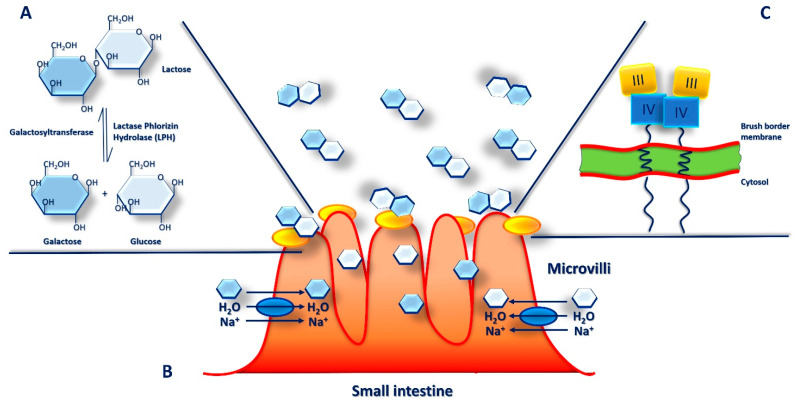
Intestinal digestion and absorption of lactose. The β-galactosidase lactase phlorizin hydrolase (LPH) breaks down lactose into glucose and galactose (white and blue diamonds) in the brush border microvilli (BBM) on the apical surface of the small intestine enterocytes (**A**). Here, after hydrolysis by LPH (yellow oval), monosaccharides are actively transported into enterocytes by the Na^+^/glucose co-transporter (blue oval) together with H_2_O molecules, rapidly absorbed into the surrounding capillaries by facilitated diffusion and transported in the bloodstream (**B**). LPH is synthesized as monomeric pro-LPH, which consists of four domains (I–IV), proteolytically activated first in the endoplasmic reticulum and in the Golgi apparatus of the enterocytes for cleavage of domains I and then sorted to BBM, where also domains II are cleaved to final mature LPH form (**C**).

In mammalian milk, lactose is a major component, the main carbohydrate and energy source as well as an important constituent of human diet. Lactose is a disaccharide made up of two monosaccharides, glucose and galactose, linked together by a β-1-4 glycosidic bond. To take benefit from lactose contained in milk and be easily absorbed from the small intestine, mammals have to first hydrolyze lactose into these two monosaccharides. In humans, hydrolysis of such bond requires a specific enzyme, a β-galactosidase called lactase-phlorizin hydrolase (LPH) (Figure 4A). The activity of intestinal lactase has a higher peak at birth, and then it reduces progressively after weaning, although it can persist also in adulthood [95,96]. In particular, it was observed that in the fetal intestine, lactase activity can be found at 8 weeks of gestation increasing considerably until 40 weeks of gestation.

Moreover, its activity increases markedly after the first feeding, with human milk reaching about totally efficiency in the first 5 days of life. In addition, it was proved that preterm infants receiving early enteral feeding gain a fully enzyme activity compared to those who delay in feeding and that breastfed infants show higher lactase levels than infants who received infant formula at 10 days of life [96]. In the small intestine, on the brush border of villi, lactose is the main substrate of LPH where it is hydrolyzed and absorbed (Figure 4B). LPH, which crosses the enterocytes apical membrane, consists of two identical extracellular polypeptide chains and a short intracytoplasmic portion (Figure 4C). However, LPH is a multifunctional enzyme with several substrates and, in addition to lactose, it can also hydrolyze cellotriose, lactosylceramide, flavonoid glucosides, cellobiose, and phlorizin.

Lactose is cleaved into the monosaccharides, glucose and galactose, which are actively transported into enterocytes by the sodium/glucose co-transporter (SGLT1) and by the second facilitative transporter (GLUT2) when their concentrations become higher. Subsequentially, the glucose reaches the circulatory system by the capillaries [85].

Globally, approximately 70% of the adult population among different countries is unable to digest lactose, the sugar present in milk, completely. This condition, also called lactose malabsorption (LM), is mainly due to limited expression of lactase enzyme in the small intestine and known as lactase deficiency (LD) which may lead to LI. People with LI show symptoms such as bloating, gas and diarrhea or dyspepsia following lactose intake as well as it may also lead to inflammatory cell changes in the colonic mucosa. However, individuals suffering from LI might manifest also extra-intestinal symptoms, based on the amount of lactose introduced with the diet as well as on the lactase activity [97,98].

On the other hand, although the main LI treatment is a lactose-restricted diet, lactase supplementation, fermented dairy products and use of probiotics and prebiotics to induce colon microbiota recovery may reduce or treat the symptomatology [86,87,99]; however, symptoms often persist in LI patients, even if the aforementioned precautions are taken. For this reason, new treatments, such as use of “selected” probiotics, are always being developed. In this scenario, Vitellio et al. highlighted the importance of Bifidobacterium longum and *Lactobacillus rhamnosus* and vitamin B6 to mitigate gut dysbiosis and the related symptoms in LI patients with persistent functional gastrointestinal symptoms [100].

Several studies show the correlation between LI and some other gastrointestinal disorders such as irritable bowel syndrome (IBS), celiac disease (CD), non-celiac gluten sensitivity (NCGS) [101,102,103,104] and small intestinal bacterial overgrowth (SIBO) [105]. In these cases, a lactose-restricted diet is often recommended, although it can potentially result in some nutrient deficiencies and complications such as bone health. In fact, LI may predispose individuals to low calcium intake and vitamin deficiency with consequent predisposition to osteoporosis as well as other gastrointestinal diseases, including like Crohn’s disease and ulcerative colitis, are risk factors for osteoporosis [106,107,108].

LI is characterized by specific signs and symptoms, including bloating, abdominal pain, and diarrhea, caused by lactose intake in individuals with LM. However, individuals with LM do not always develop LI. For this reason, although LM is a necessary precondition for LI, it is not sufficient since LM can have primary or secondary causes. In addition, there is no matching between symptomatology and a positive test for LI [109]. Lactase non-persistence (LNP) is the primary cause of LM, due to reduction in expression of lactase levels during the first two decades of life, while lactase persistence (LP) derives from specific mutations. Moreover, recent research showed that LNP may be an ancestral condition that following Mendelian genetics and its prevalence is based not only on individual genetics but also on region of origin, with higher frequency in individuals of Asian, African, and South American, and lower in those of northwestern and Indian northern European origin [85,109]. Different causes lead to LD, which distinguish each of the distinct four types:

***Primary Lactase Deficiency***: defined also as adult-type LD, is a common autosomal recessive inherited condition characterized by a progressive reduction in lactase activity; in fact, it results from a regulated change in the lactase gene expression during the life span [90,110,111].
***Secondary Lactase Deficiency***: is a transient condition caused by damage to the intestinal epithelium following to several diseases including infections, small bowel bacterial overgrowth, AIDS, malnutrition, Crohn’s disease, IBS, antibiotic usage, celiac disease, radiation/chemotherapy, rotavirus gastroenteritis, food allergy, and antibiotic usage. However, the reduction in lactase activity is transient and reversible and improves once the intestinal damage is resolved [90,110,111].
***Congenital Lactase Deficiency***: is a pediatric autosomal recessive inherited disease that affects infants and leads to a reduced or absent lactase activity at birth. This is an extremely rare disorder, resulting in severe and potentially lethal symptoms with serious difficulties in growth and development after birth [90,110,111].
***Developmental Lactase Deficiency***: is a condition observed in premature neonates born between 28 and 37 weeks of gestation. These neonates generally have incomplete development of the enzyme or insufficient intestinal lactase activity. This condition can improve with age and as intestine matures with feeding [90,110,111].

Nowadays, to confirm the different types of LD with LI symptomatology, diagnostic clinical tests as well as histological exams are available such as blood, biopsy, genetic, and breath tests (Table 1).

The LPH enzyme is encoded by the LCT gene, which is located on chromosome 2q21 and consists of 17 exons extended for approximately 49 kb and originating from a mRNA of slightly more than 6 kb. The monomeric pro-LPH form consists of four domains (I–IV); following proteolytic cleavage, the final mature LPH protein is characterized by two domains (III–IV) (Figure 4C).

LPH is expressed in the small intestine exclusively. It is known that LP could be caused by mutations; in particular five or more single-nucleotide polymorphisms (SNPs) can occur in a regulatory region, located upstream of the LCT gene, called MCM6 (minichromosome maintenance complex component 6). Although MCM6 is a regulatory enhancer of LCT and that these two genes are close together, some evidence indicates that they are regulated in an independent manner [112,113].

Several studies highlight the genetic basis to identify human lactase phenotypes of both in LP and LNP. Until today, numerous genetic variants responsible for LNP and LP were identified. Among all polymorphisms, the −13910:C>T (rs4988235) variant is the most widespread in some parts of Europe, while other variants including −14009:T>G (rs869051967), −13907:C>G (rs41525747), −14010:G>C (rs145946881) and −13915:T>G (rs41380347) are found in Africa and the Middle East, with mutable frequency and others have low or rare frequencies. Moreover, SNP frequencies differ not only between different countries but also between subpopulations within the same geographical area. −13910:C>T (rs4988235) and –22018:G>A (rs182549) are the more studied variants, are present in many ethnic groups and show complete co-segregation with LNP/LP in Europeans. Moreover, the relation between these two polymorphisms and their incidence in hypolactasia are well known [85,112,113,114].

The polymorphic variant −13910:C>T (rs4988235) arises from the CC, CT or TT genotype. While the CC genotype is a predictor of low intestinal lactase expression, the TT genotype is a predictor of high lactase expression. On the other hand, the CT genotype indicates an intermediate amount of lactase expression, sufficient to digest lactose [114]. The main variants are summarized in Table 2.

Several variants have been characterized and associated with congenital lactase deficiency. Marten et al. have studied the effects of some LCT variants frequently detected in congenital lactase deficiency and have proven that, analyzing the cellular LCT levels, these mutants are totally transport incompetent, enzymatically inactive and some of which readily degraded [115]. Moreover, Hoang et al. have observed and suggest an association between maternal LD, with the rs4988235 genotype, and risk of neural tube defects in the mother’s progeny among Caucasians and Hispanics [116].

The polymorphic variant −13910:C>T seems to also be correlated to nutritional deficiencies such as vitamin D and calcium. In a preliminary study, Kowalówka et al. observed that young Polish adults with polymorphic variant −13910:C>T showed lower levels of vitamin D and calcium in LNP subjects compared with those with the −13910:T>T variant [117]. Moreover Domżał-Magrowska et al., based on the results of the hydrogen breath test, observed no statistically significant differences between IBS patients who had the −13910:C>T and −22018:G>A polymorphism compared with the control group reporting hypolactasia [118].

## 8. Omics Tools: Nutrigenetics and Epigenetics Approaches for Lactose Intolerance Management

The omics technologies, such as genomics, epigenomics and metabolomics, shedding some light on the complex interactions between our body and nutrients, can help us to diagnose and treat LI [119,120]. Polymorphism frequency is analyzed in a given population through nutrigenetics and predicts our metabolic response according to our individual genotype [121]. To date, only a few genetic tests are offered by clinicians in order to translate and integrate genetic information into a personalized healthier diet for their patients [120]. The polymorphisms in regulatory sequences of the *LCT* gene most frequently associated with LI in the European population are −13910:C>T and −22018:G>A and their genetic tests are available in Europe [122,123].

Nevertheless, only these SNPs cannot be used as a worldwide diagnostic tool, because they represent a small percentage of all the twenty-three LP variants to date identified in the *MCM6* gene in different geographic regions and populations [112,122,124]. Direct-to-consumer genetic testing (DTC-GT) is currently available to customers by specialized companies giving access to nutrigenetic information without the help of specialized health personnel [125], allowing them to investigate their genetic susceptibility in order to make a diet tailored to their needs [126]. Customers can buy these tests on-line together with sample collection kits. Then, the collected sample (saliva or cheek swab) is sent to the company for analysis and the results will be finally sent to customers by mail or online account, providing information on LI, or other monogenic disorders, risk and selling personalized feeding strategies, dietary supplements and/or physical training programs [126].

Unfortunately, it is very easy to make mistakes in genetic data interpretation, thus leading to unnecessary dietary restrictions making the advice of a health professional expert very useful [127,128]. Self-reported LI and genetic test results are often unrelated and people choose to avoid foods containing lactose only due to symptoms after eating them [97,129,130]. In addition, a recent report on DTC-GT relative to LI highlighted the presence of numerous false positives (40%) among the genetic results, making it further difficult to establish their scientific reliability [125,131]. Thus, it becomes increasingly clear that DTC-GT have to be improved in sensitivity and specificity with the aim of providing more accurate information on the *LCT* gene variant tested and its correlation with LI or other diseases, so as to prevent the consumer from being subjected to unnecessary diets and restrictions.

In addition to the existence of *LCT* gene variants, epigenetics processes could be responsible for the onset of LI in adulthood as well as for the progressive reduction in lactase levels during childhood [112,132]. To confirm this hypothesis, recently, Labrie and colleagues reported that when epigenetic changes, like DNA methylation, accumulate in senescent cells they modify the expression of lactase depending on the genetic variants that regulate the expression of the *LCT* gene [133]. In the specific, DNA methylation means addition of a methyl group to the DNA cytosine with a consequent repression of gene expression. LNP individuals are characterized by methylation in both the *MCM6* and *LCT* genes, resulting in low *LCT* expression levels and the occurrence of LI with age, while LP individuals do not develop LI symptoms with age, because their DNA seems not to undergo epigenetic changes, such as cytosine methylation [133].

Leseva et al., through an epigenome-wide approach, characterized the position in the *LCT* promoter with an inverse correlation between methylation levels and lactase enzymatic activity, suggesting that using epigenetic and genetic data information together can be more helpful in detecting LI than using them individually [134]. They also reported a correlation between changes in DNA methylation at the *LCT* promoter and lactase-mRNA levels in intestinal cells, as they found a significant reduction in its levels with age only in LNP subjects. In addition, they also reported that in these individuals, further epigenetics changes occurring in histone proteins, altering the structure of chromatin and DNA accessibility, could contribute to *LCT* gene repression [133]. Taken together, these findings have brought to light how LI development may result from the coexistence in time of genetic polymorphisms and epigenetic modifications.

Unfortunately, little is known about a possible correlation between weaning and reduction in lactase activity, probably due, as demonstrated in rats, to a reduction in transcription levels of the gene that codifies for LPH enzyme, process absolutely unrelated to stopping milk intake [135,136]. Experimental evidence rather suggests not only a transcriptional regulation for lactase activity in humans occurring after weaning but also an additional regulation at the post-translational level, even though it has yet to be understood and demonstrated [137]. Furthermore, epigenetic modifications too may be involved in the regulation of lactase activity in infants who are weaned.

## 9. Precision Nutrition through Metabolomics Approaches

Metabolomics approaches enable us to make precise chemical analyses of up to a thousand of metabolites, to investigate how they are affected by gene mutations causing proteins changes and to characterize metabolic processes that underlie several pathologies with the goal to find new functional food biomarkers, or therapeutic targets, of disease, with a leading role in our metabolism [138]. 

We can use metabolomics approaches to quantify the serum levels of lactose after dairy intake, as reported by Pimentel et al. [139].

Generally, lactose is not absorbed in the form of disaccharide by intestinal cells due to the lack of appropriate carriers, but sometimes little traces of it are found in blood and urine after eating [139]. In addition, metabolomics analysis allows us to identify galactitol and galactonate, two metabolites produced by hepatic galactose metabolism, whose serum and urine postprandial levels increase in LP, but not in LNP, subjects who ingested lactose, being proposed as novel and non-invasive lactose digestion test for LI screening [140]. Therefore, metabolomics help us to analyze the effect of single nutrients, and their metabolic products, on individual’s health, leading to comprehend how the same foods are metabolized differently by different individuals in healthy or unhealthy conditions, such as LI, and so to plan a personalized nutrition or PN program [141].

Biological variability between individuals in response to nutrition is the basis of PN [142]. This area of nutrition focuses on the effects of the nutrients over the genome, proteome and metabolome [143], trying to clarify how gene expression may be affected by nutrients introduced with the diet, in order to promote well-being and health, to prevent diseases, especially reducing chronic disease incidence, and to increase life expectancy [144]. Nutrigenomics, if applied to LI research field, could help with the identification of groups of individuals, among different populations, characterized by the high prevalence of LNP, suggesting dietary restrictions, ensuring optimal nutrients and daily energy intake, with some kind of guidelines promoting healthy eating or “Dietary Reference Values (DRVs) [145].

PN improves public health nutrition through defining and identifying groups of individuals with specific distinct dietary requirements as for LNP subjects with a lower calcium intake when compared to LP subjects [146]. This can be overcome by consuming dairy products, with or without lactose, with adaptation strategies or promoting alternative dietary calcium sources for this subpopulation.

PN is used to create dietary strategies different for each group of individuals on the basis of the different individual lactose tolerance threshold and taking into account their needs in terms of specific nutrients intake. Finally, nutrigenomics research is crucial to define lactose malabsorption from other physiological processes that could determine the appearance of adverse gastrointestinal consequences in LI population.

Omics technologies help us to investigate why and how the individual responses to diet are different and the mechanisms that regulate these differences. Thus, PN replaces the “one size fits all” approach, offering individual dietary interventions through the integration of nutrigenomic data together with clinical parameters and microbiota profiles to design a customized diet.

The right LI approach includes a lactose-free, or low-lactose diet, colon microbiome adaptation, using specific probiotic strains with β-galactosidase enzymatic activity, and oral lactase enzyme replacement [147].

Indeed, while individuals with a milk allergy need to remove all dairy products from the diet, not all those with LI have to fully eliminate lactose. Sometimes, reducing its consumption until the symptoms disappear is enough. Therefore, the dietetic approach plays a crucial role in the management of LI. Individuals with LI are usually treated with a lactose-free diet to reduce symptom manifestations [147], although the avoidance of all dairy products is no longer recommended today, as the majority of LI patients can tolerate up to 5 g of lactose, approximately 100 mL of milk, per single dose. In addition, the tolerance threshold increases if the lactose is consumed together with other nutrients. In this context, “Nutrition Guidelines for Lactose Intolerance” have to be followed, to ensure that LI subjects choose products containing the appropriate amount of lactose, without excluding all the dairy products from the diet [148,149].

Data obtained on LI subjects’ nutrient intake, compared to those from lactose-tolerant individuals, show that they consume lower amounts of calcium, with average intake ranging from 388 to 739 mg a day, below the Recommended Dietary Allowance (RDA) of 1000 mg a day [106], with a major risk of developing a calcium deficiency and compromising bone health.

Therefore, it is crucial to ensure an appropriate calcium intake at each age to maintain a healthy skeleton, especially in LI individuals [110]. In addition to calcium, vitamin D, vitamin A, potassium, zinc, and magnesium present in dairy products are also important nutrients for the formation of healthy and strong bones, as suggested by Heaney and colleagues in their review [150]. The common scientific opinion agrees that dairy foods fulfill all the nutritional requirements for a proper bone status and that it is challenging to reach the recommended calcium intake without the use of dairy products.

In most cases, reducing the consumption of, or avoiding lactose-containing foods and drinks, and replacing them with lactose-free alternatives is sufficient to control the unpleasant symptoms of intolerance. There are several alternative foods and drinks, both artificial and natural, able to replace milk and dairy products, including lactose-free dairy products and plant-based milk food [151]. In this regard, there are many lactose-free kinds of cheese available on the market, such as Grana Padano and Parmigiano Reggiano. They are naturally “lactose free” and rich in nutritious substances, especially protein, calcium and phosphorus, thanks to their hard-maturation process (the longer a cheese has been matured, the less lactose remains in the final product); therefore, the low lactose concentration in both of them (<0.1 g/100 g) can be easily tolerated by most individuals suffering from primary LI [152].

Moreover, also modulating the composition of gut microbiota seems to be a promising therapeutic strategy for LI, along with progressive and regular intake of lactose as well as the ingestion of GOS in combination with pre- and probiotics specific for each microbiota profile [84,95,153].

Most LI individuals can eat yogurt without exhibiting typical symptoms; moreover, yogurt consumption is suggested as a suitable dietary strategy to reach the recommended daily intake of calcium for LI individuals [154,155]. In particular, some yogurt culture microorganisms, such as *L. delbrueckii* subsp. *bulgaricus* and *S. thermophilus*, produce β-galactosidase as part of their lactose utilization pathway, promoting lactose digestion [156,157]. Furthermore, lactose found in yogurt seems to be better digested due to the decreased transit time of a viscous meal, like yogurt, rather than a liquid one, like milk. Any extra lactase in the small intestine has more time to digest lactose, reducing intolerance symptoms [146,156,158]. Lactose digestion can also be improved by the use of particular species of bacteria in the yogurt-making process [146,158], although the most reliable solution remains the complete enzymatic digestion of lactose in yogurt by incubating the milk with lactase before pasteurization (yogurt from hydrolyzed milk) or adding the lactase together with the culture and fermentation at once (co-hydrolysis). These are only few examples of how diet can be supplemented so that our body does not suffer from nutritional deficiencies due to the total or partial deprivation of foods containing lactose, depending on our lactose tolerance threshold.

The progress made by the research on gene–diet interactions and the identification of SNPs that modulate the individual response to specific nutrients, and their possible influence on disease risk, is helpful to develop PN recommendations for the management of monogenic condition caused by single-gene mutation and its correlation to single diet component ingestion, rather than for polygenic disorders [119,120,159]. Indeed, when multiple SNPs are associated with environmental factors, such as for polygenic disorders like cancer, obesity or metabolic syndrome, developing a genetically tailored diet and translating genetic research into PN can be very difficult [120].

LI, although its onset may be due to the interaction of many polymorphisms still uncharacterized affecting *LCT* gene expression with epigenetic changes and gut microbiota alterations, is considered a monogenic disorder that could be easily treated through a lactose-free diet or through the ingestion of a reduced amount of lactose, providing a simple model for the implementation of genetic research within PN [120]. LI DTC-GT tests are often expensive (from USD 100 up to USD 200) and not covered by health insurance, not being available to all individuals or societies. Thus, they could be distributed in pharmacies with the aim to overcome the problems of accessibility of PN to all LI individuals, even the most socioeconomically disadvantaged, providing genetic information integrated by artificial intelligence and diet-related tailor-made recommendations in the form of 24 h smartphone apps, creating a real lifestyle market for LI [143].

## 10. Conclusions

Omics data management is very complex: genetic results have to be well interpreted and integrated into effective and practical PN recommendations [120], often requiring the involvement of a trained medical staff.

Everything is more complicated with the recent findings on epigenetics and gut microbiota involvement in LI, leading to the understanding that LI cannot be any longer considered to be caused by a single penetrant genetic polymorphism. Furthermore, another challenge occurring when we try to apply PN for the management of LI is the lack of regulation of certain food labels, there being no agreement on a specific lactose-free or reduced-lactose label since there is no precise cut-off value for establishing a lactose-free labeling policy, except for infant formula [97,147]. Thus, consumers are not adequately protected, and they lose the personal freedom of choosing foods to eat because the amount of lactose present is not precisely declared [160,161].

Finally, LI is often considered to be something different from a normal state and the perception of this condition from each individual has a psychological impact on quality-of-life scores [162], with a higher risk of depression or anxiety [163]. Thus, implementing PN should take into account the need for an increased availability of diagnostic tools, personalized dietary recommendations and psychological support.

## Figures and Tables

**Table 1:** Diagnostic tests currently used for detecting LM/LI.

TEST	APPLICATION	TEST PRINCIPLE
Hydrogen breath test (HBT)	Test of choice for the diagnosis of LM/LI	Detection of increase H_2_ in expiratory air after lactose intake
Lactose tolerance test	LM epidemiology, low sensitivity and specificity	Increase in blood sugar after lactose intake
Lactase activity of jejunum	If gastroscopy is carried out for other investigations, invasive and expensive	Evaluation of lactase enzymatic activity in duodenal biopsy sample
Genetic test	LD/LNP	Test for detection of −13910:C>T polymorphism
Serum gaxilose or urine galactose test	Used for the diagnosis of small intestine diseases (intestinal malabsorption)	Evaluation of D-xylose in plasma or galactose in urine after cleavage by lactase of 4-galactosylxylose oral administration

**Table 2:** SNPs, variants and the geographic region of identification.

SNP	VARIANT	GEOGRAPHIC REGION
rs4988235	−13910:C>T	Europe
rs869051967	−14009:T>G	Middle East
rs4988236	−13908:G>A	Far East
rs773131166	−13914:C>T	East Europe
rs41380347	−13915:A>C	Middle East
rs41525747	−13907:G>C	Middle East
rs820486563	−14009:T>G	Ethiopia and Sudan
rs182549	–22018:G>A	Europe
rs41456145	−13913:A>G	Africa
rs145946881	−14010:C>G	Kenya, Tanzania and South Africa

## References

[1] Pereira P.C. (2014). Milk nutritional composition and its role in human health. Nutrition.

[2] Heinig M.J., Nommsen L.A., Peerson J.M., Lonnerdal B., Dewey K.G. (1993). Energy and protein intakes of breast-fed and formula-fed infants during the first year of life and their association with growth velocity: The DARLING Study. Am. J. Clin. Nutr..

[3] Khan I.T., Nadeem M., Imran M., Ayaz M., Ajmal M., Ellahi M.Y., Khalique A. (2017). Antioxidant capacity and fatty acids characterization of heat-treated cow and buffalo milk. Lipids Health Dis..

[4] Leduc A., Souchet S., Gele M., Le Provost F., Boutinaud M., Pascottini O., Carvalho M., Schyndel S., Ticiani E., Spricigo J. (2021). Effect of feed restriction on dairy cow milk production. J. Anim. Sci..

[5] Foroutan A., Guo A.C., Vazquez-Fresno R., Lipfert M., Zhang L., Zheng J., Badran H., Budinski Z., Mandal R., Ametaj B.N. (2019). Chemical composition of commercial cow’s milk. J. Agric. Food Chem..

[6] Leischner C., Egert S., Burkard M., Venturelli S. (2021). Potential protective protein components of cow’s milk against certain tumor entities. Nutrients.

[7] Wang A., Duncan S.E., Knowlton K.F., Ray W.K., Dietrich A.M. (2016). Milk protein composition and stability changes affected by iron in water sources. J. Dairy Sci..

[8] Dyrda-Terniuk T., Pryshchepa O., Rafińska K., Kolankowski M., Gołębiowski A., Gloc M., Dobrucka R., Kurzydłowski K., Pomastowski P. (2023). Immobilization of silver ions onto casein. Colloids Surf. A Physicochem. Eng. Asp..

[9] Thorning T.K., Raben A., Tholstrup T., Soedamah-Muthu S.S., Givens I., Astrup A. (2016). Milk and dairy products: Good or bad for human health? An assessment of the totality of scientific evidence. Food Nutr. Res..

[10] Carter B., Cheng N., Kapoor R., Meletharayil G., Drake M. (2021). Invited review: Microfiltration-derived casein and whey proteins from milk. J. Dairy Sci..

[11] Carter B., DiMarzo L., Pranata J., Barbano D.M., Drake M. (2021). Efficiency of removal of whey protein from sweet whey using polymeric microfiltration membranes. J. Dairy Sci..

[12] Fernández-Rico S., Mondragón A.D.C., López-Santamarina A., Cardelle-Cobas A., Regal P., Lamas A., Ibarra I.S., Cepeda A., Miranda J.M. (2022). A2 Milk: New Perspectives for Food Technology and Human Health. Foods.

[13] Ul Haq M.R., Kapila R., Saliganti V. (2014). Consumption of β-casomorphins-7/5 induce inflammatory immune response in mice gut through Th2 pathway. J. Funct. Foods.

[14] Jung T.H., Hwang H.J., Yun S.S., Lee W.J., Kim J.W., Ahn J.Y., Jeon W.M., Han K.S. (2017). Hypoallergenic and physicochemical properties of the A2 beta-casein fraction of goat milk. Korean J. Food Sci. Anim. Resour..

[15] Kim J., Paik H.-D., Yoon Y.-C., Park E. (2013). Whey protein inhibits iron overload-induced oxidative stress in rats. J. Nutr. Sci. Vitaminol..

[16] Zeng B., Chen T., Xie M.-Y., Luo J.-Y., He J.-J., Xi Q.-Y., Sun J.-J., Zhang Y.-L. (2019). Exploration of long noncoding RNA in bovine milk exosomes and their stability during digestion in vitro. J. Dairy Sci..

[17] Feng X., Chen X., Zheng X., Zhu H., Qi Q., Liu S., Zhang H., Che J. (2021). Latest trend of milk derived exosomes: Cargos, functions, and applications. Front. Nutr..

[18] Contarini G., Povolo M. (2013). Phospholipids in milk fat: Composition, biological and technological significance, and analytical strategies. Int. J. Mol. Sci..

[19] Ortega-Anaya J., Jiménez-Flores R. (2019). Symposium review: The relevance of bovine milk phospholipids in human nutrition—Evidence of the effect on infant gut and brain development. J. Dairy Sci..

[20] Stonehouse W., Klingner B., McJarrow P., Fong B., O’callaghan N. (2020). Exploring in vivo dynamics of bovine milk derived gangliosides. Nutrients.

[21] Benbrook C.M., Butler G., Latif M.A., Leifert C., Davis D.R. (2013). Organic production enhances milk nutritional quality by shifting fatty acid composition: A United States–wide, 18-month study. PLoS ONE.

[22] Warstedt K., Furuhjelm C., Fälth-Magnusson K., Fagerås M., Duchén K. (2016). High levels of omega-3 fatty acids in milk from omega-3 fatty acid-supplemented mothers are related to less immunoglobulin E-associated disease in infancy. Acta Paediatr..

[23] Kasapidou E., Basdagianni Z., Papatzimos G., Papadopoulos V., Tsiftsi E., Neki I., Nigianni P.-A., Mitlianga P. (2023). Chemical composition, antioxidant profile and physicochemical properties of commercial non-cocoa-and cocoa-flavoured plant-based milk alternatives. Eur. Food Res. Technol..

[24] Gaucheron F. (2005). The minerals of milk. Reprod. Nutr. Dev..

[25] Sorensen M.D. (2014). Calcium intake and urinary stone disease. Transl. Androl. Urol..

[26] Heravi A.S., Michos E.D. (2019). Vitamin D and calcium supplements: Helpful, harmful, or neutral for cardiovascular risk?. Methodist DeBakey Cardiovasc. J..

[27] Huo X., Clarke R., Halsey J., Jackson R., Lehman A., Prince R., Lewis J., Baron J.A., Kroger H., Sund R. (2023). Calcium Supplements Treatment Trialists’ Collaboration. Calcium Supplements and Risk of CVD: A Meta-Analysis of Randomized Trials. Curr. Dev. Nutr..

[28] Drouin-Chartier J.P., Brassard D., Tessier-Grenier M., Côté J.A., Labonté M.È., Desroches S., Couture P., Lamarche B. (2016). Systematic Review of the Association between Dairy Product Consumption and Risk of Cardiovascular-Related Clinical Outcomes. Adv. Nutr..

[29] Gudi S.K. (2021). Dairy consumption and risk of type-2 diabetes: The untold story. Ann. Pediatr. Endocrinol. Metab..

[30] Gil H., Chen Q.-Y., Khil J., Park J., Na G., Lee D., Keum N. (2022). Milk intake in early life and later cancer risk: A meta-analysis. Nutrients.

[31] Arafat H.M., Omar J., Shafii N., Naser I.A., Al Laham N.A., Muhamad R., Al-Astani T.A.D., Shaqaliah A.J., Shamallakh O.M., Shamallakh K.M. (2023). The association between breast cancer and consumption of dairy products: A systematic review. Ann. Med..

[32] Kumar A., Chinnathambi S., Kumar M., Pandian G.N. (2023). Food Intake and Colorectal Cancer. Nutr. Cancer.

[33] Givens D. (2020). MILK Symposium review: The importance of milk and dairy foods in the diets of infants, adolescents, pregnant women, adults, and the elderly. J. Dairy Sci..

[34] Corsello A., Pugliese D., Gasbarrini A., Armuzzi A. (2020). Diet and Nutrients in Gastrointestinal Chronic Diseases. Nutrients.

[35] Schoemaker A.A., Sprikkelman A.B., Grimshaw K.E., Roberts G., Grabenhenrich L., Rosenfeld L., Siegert S., Dubakiene R., Rudzeviciene O., Reche M. (2015). Incidence and natural history of challenge-proven cow’s milk allergy in European children--EuroPrevall birth cohort. Allergy.

[36] Nwaru B.I., Hickstein L., Panesar S.S., Roberts G., Muraro A., Sheikh A. (2014). Prevalence of common food allergies in Europe: A systematic review and meta-analysis. Allergy.

[37] Katz Y., Rajuan N., Goldberg M.R., Eisenberg E., Heyman E., Cohen A., Leshno M. (2010). Early exposure to cow’s milk protein is protective against IgE-mediated cow’s milk protein allergy. J. Allergy Clin. Immunol..

[38] García-Ara M.C., Boyano-Martínez M.T., Díaz-Pena J.M., Martín-Muñoz M.F., Martín-Esteban M. (2004). Cow’s milk-specific immunoglobulin E levels as predictors of clinical reactivity in the follow-up of the cow’s milk allergy infants. Clin. Exp. Allergy J. Br. Soc. Allergy Clin. Immunol..

[39] Fiocchi A., Terracciano L., Bouygue G.R., Veglia F., Sarratud T., Martelli A., Restani P. (2008). Incremental prognostic factors associated with cow’s milk allergy outcomes in infant and child referrals: The Milan Cow’s Milk Allergy Cohort study. Ann. Allergy Asthma Immunol..

[40] Skripak J.M., Matsui E.C., Mudd K., Wood R.A. (2007). The natural history of IgE-mediated cow’s milk allergy. J. Allergy Clin. Immunol..

[41] Fiocchi A., Schünemann H.J., Brozek J., Restani P., Beyer K., Troncone R., Martelli A., Terracciano L., Bahna S.L., Rancé F. (2010). Diagnosis and Rationale for Action Against Cow’s Milk Allergy (DRACMA): A summary report. J. Allergy Clin. Immunol..

[42] Tsabouri S., Douros K., Priftis K.N. (2014). Cow’s milk allergenicity. Endocr. Metab. Immune Disord. Drug Targets.

[43] Sampson H.A. (1999). Food allergy. Part 1: Immunopathogenesis and clinical disorders. J. Allergy Clin. Immunol..

[44] Vickery B.P., Chin S., Burks A.W. (2011). Pathophysiology of food allergy. Pediatr. Clin. N. Am..

[45] Luyt D., Ball H., Makwana N., Green M.R., Bravin K., Nasser S.M., Clark A.T. (2014). BSACI guideline for the diagnosis and management of cow’s milk allergy. Clin. Exp. Allergy J. Br. Soc. Allergy Clin. Immunol..

[46] Caldwell J.M., Paul M., Rothenberg M.E. (2017). Novel immunologic mechanisms in eosinophilic esophagitis. Curr. Opin. Immunol..

[47] Giannetti A., Toschi Vespasiani G., Ricci G., Miniaci A., di Palmo E., Pession A. (2021). Cow’s Milk Protein Allergy as a Model of Food Allergies. Nutrients.

[48] Sicherer S.H., Sampson H.A. (2018). Food allergy: A review and update on epidemiology, pathogenesis, diagnosis, prevention, and management. J. Allergy Clin. Immunol..

[49] Savage J.H., Lee-Sarwar K.A., Sordillo J., Bunyavanich S., Zhou Y., O’Connor G., Sandel M., Bacharier L.B., Zeiger R., Sodergren E. (2018). A prospective microbiome-wide association study of food sensitization and food allergy in early childhood. Allergy.

[50] Roduit C., Frei R., Depner M., Schaub B., Loss G., Genuneit J., Pfefferle P., Hyvärinen A., Karvonen A.M., Riedler J. (2014). Increased food diversity in the first year of life is inversely associated with allergic diseases. J. Allergy Clin. Immunol..

[51] Perkin M.R., Logan K., Marrs T., Radulovic S., Craven J., Flohr C., Lack G. (2016). Enquiring About Tolerance (EAT) study: Feasibility of an early allergenic food introduction regimen. J. Allergy Clin. Immunol..

[52] Venter C., Maslin K., Holloway J.W., Silveira L.J., Fleischer D.M., Dean T., Arshad S.H. (2020). Different Measures of Diet Diversity During Infancy and the Association with Childhood Food Allergy in a UK Birth Cohort Study. J. Allergy Clin. Immunol. Pract..

[53] Koplin J.J., Allen K.J., Gurrin L.C., Peters R.L., Lowe A.J., Tang M.L., Dharmage S.C. (2013). The impact of family history of allergy on risk of food allergy: A population-based study of infants. Int. J. Environ. Res. Public. Health.

[54] Hill D.J., Hosking C.S. (2004). Food allergy and atopic dermatitis in infancy: An epidemiologic study. Pediatr. Allergy Immunol..

[55] Boyano-Martínez T., García-Ara C., Pedrosa M., Díaz-Pena J.M., Quirce S. (2009). Accidental allergic reactions in children allergic to cow’s milk proteins. J. Allergy Clin. Immunol..

[56] Burris A.D., Burris J., Järvinen K.M. (2020). Cow’s milk protein allergy in term and preterm infants: Clinical manifestations, immunologic pathophysiology, and management strategies. NeoReviews.

[57] Toro-Monjaraz E.M., Fonseca-Camarillo G., Zárate-Mondragón F., Montijo-Barrios E., Cadena-León J., Avelar-Rodríguez D., Ramírez-Mayans J., Cervantes-Bustamante R., Yamamoto-Furusho J.K. (2021). Differential cytokine expression in the duodenum and rectum of children with non-immunoglobulin e-mediated cow’s milk protein allergy. Dig. Dis. Sci..

[58] Vitaliti G., Cimino C., Coco A., Praticò A.D., Lionetti E. (2012). The immunopathogenesis of cow’s milk protein allergy (CMPA). Ital. J. Pediatr..

[59] Athie-Morales V., Smits H.H., Cantrell D.A., Hilkens C.M. (2004). Sustained IL-12 signaling is required for Th1 development. J. Immunol..

[60] Paajanen L., Kokkonen J., Karttunen T.J., Tuure T., Korpela R., Vaarala O. (2006). Intestinal cytokine mRNA expression in delayed-type cow’s milk allergy. J. Pediatr. Gastroenterol. Nutr..

[61] Veres G., Westerholm-Ormio M., Kokkonen J., Arato A., Savilahti E. (2003). Cytokines and adhesion molecules in duodenal mucosa of children with delayed-type food allergy. J. Pediatr. Gastroenterol. Nutr..

[62] Paajanen L., Vaarala O., Karttunen R., Tuure T., Korpela R., Kokkonen J. (2005). Increased IFN-γ secretion from duodenal biopsy samples in delayed-type cow’s milk allergy. Pediatr. Allergy Immunol..

[63] Monin L., Gaffen S.L. (2018). Interleukin 17 family cytokines: Signaling mechanisms, biological activities, and therapeutic implications. Cold Spring Harb. Perspect. Biol..

[64] Clayton F., Fang J.C., Gleich G.J., Lucendo A.J., Olalla J.M., Vinson L.A., Lowichik A., Chen X., Emerson L., Cox K. (2014). Eosinophilic esophagitis in adults is associated with IgG4 and not mediated by IgE. Gastroenterology.

[65] Schuyler A.J., Wilson J.M., Tripathi A., Commins S.P., Ogbogu P.U., Kruzsewski P.G., Barnes B.H., McGowan E.C., Workman L.J., Lidholm J. (2018). Specific IgG4 antibodies to cow’s milk proteins in pediatric patients with eosinophilic esophagitis. J. Allergy Clin. Immunol..

[66] Aalberse R.C., Platts-Mills T.A., Rispens T. (2016). The developmental history of IgE and IgG4 antibodies in relation to atopy, eosinophilic esophagitis, and the modified TH 2 response. Curr. Allergy Asthma Rep..

[67] Pratelli G., Tamburini B., Carlisi D., De Blasio A., D’Anneo A., Emanuele S., Notaro A., Affranchi F., Giuliano M., Seidita A. (2023). Foodomics-Based Approaches Shed Light on the Potential Protective Effects of Polyphenols in Inflammatory Bowel Disease. Int. J. Mol. Sci..

[68] Di Tommaso N., Gasbarrini A., Ponziani F.R. (2021). Intestinal barrier in human health and disease. Int. J. Environ. Res. Public. Health.

[69] Parada Venegas D., De la Fuente M.K., Landskron G., González M.J., Quera R., Dijkstra G., Harmsen H.J., Faber K.N., Hermoso M.A. (2019). Short chain fatty acids (SCFAs)-mediated gut epithelial and immune regulation and its relevance for inflammatory bowel diseases. Front. Immunol..

[70] Bäckhed F., Roswall J., Peng Y., Feng Q., Jia H., Kovatcheva-Datchary P., Li Y., Xia Y., Xie H., Zhong H. (2015). Dynamics and stabilization of the human gut microbiome during the first year of life. Cell Host Microbe.

[71] Eckburg P.B., Bik E.M., Bernstein C.N., Purdom E., Dethlefsen L., Sargent M., Gill S.R., Nelson K.E., Relman D.A. (2005). Diversity of the human intestinal microbial flora. Science.

[72] Arumugam M., Raes J., Pelletier E., Le Paslier D., Yamada T., Mende D.R., Fernandes G.R., Tap J., Bruls T., Batto J.-M. (2011). Enterotypes of the human gut microbiome. Nature.

[73] Turroni F., Milani C., Duranti S., Lugli G.A., Bernasconi S., Margolles A., Di Pierro F., Van Sinderen D., Ventura M. (2020). The infant gut microbiome as a microbial organ influencing host well-being. Ital. J. Pediatr..

[74] Adak A., Khan M.R. (2019). An insight into gut microbiota and its functionalities. Cell. Mol. Life Sci..

[75] del Carmen Tocaa M., Fernándezb A., Orsic M., Tabaccod O., Vinderolae G. (2022). Lactose intolerance: Myths and facts. An update. Arch. Argent. Pediatr..

[76] Gaboriau-Routhiau V., Rakotobe S., Lecuyer E., Mulder I., Lan A., Bridonneau C., Rochet V., Pisi A., De Paepe M., Brandi G. (2009). The key role of segmented filamentous bacteria in the coordinated maturation of gut helper T cell responses. Immunity.

[77] Corbett A.J., Eckle S.B., Birkinshaw R.W., Liu L., Patel O., Mahony J., Chen Z., Reantragoon R., Meehan B., Cao H. (2014). T-cell activation by transitory neo-antigens derived from distinct microbial pathways. Nature.

[78] Fernando M.R., Saxena A., Reyes J.-L., McKay D.M. (2016). Butyrate enhances antibacterial effects while suppressing other features of alternative activation in IL-4-induced macrophages. Am. J. Physiol.-Gastrointest. Liver Physiol..

[79] Albenberg L.G., Wu G.D. (2014). Diet and the intestinal microbiome: Associations, functions, and implications for health and disease. Gastroenterology.

[80] Tamburini B., La Manna M.P., La Barbera L., Mohammadnezhad L., Badami G.D., Shekarkar Azgomi M., Dieli F., Caccamo N. (2022). Immunity and nutrition: The right balance in inflammatory bowel disease. Cells.

[81] Furusawa Y., Obata Y., Fukuda S., Endo T.A., Nakato G., Takahashi D., Nakanishi Y., Uetake C., Kato K., Kato T. (2013). Commensal microbe-derived butyrate induces the differentiation of colonic regulatory T cells. Nature.

[82] Zhong Y., Priebe M.G., Vonk R.J., Huang C.-Y., Antoine J.-M., He T., Harmsen H.J., Welling G.W. (2004). The role of colonic microbiota in lactose intolerance. Dig. Dis. Sci..

[83] Azcarate-Peril M.A., Ritter A.J., Savaiano D., Monteagudo-Mera A., Anderson C., Magness S.T., Klaenhammer T.R. (2017). Impact of short-chain galactooligosaccharides on the gut microbiome of lactose-intolerant individuals. Proc. Natl. Acad. Sci. USA.

[84] Arnold J.W., Simpson J.B., Roach J., Bruno-Barcena J.M., Azcarate-Peril M.A. (2018). Prebiotics for lactose intolerance: Variability in galacto-oligosaccharide utilization by intestinal Lactobacillus rhamnosus. Nutrients.

[85] Misselwitz B., Butter M., Verbeke K., Fox M.R. (2019). Update on lactose malabsorption and intolerance: Pathogenesis, diagnosis and clinical management. Gut.

[86] Leis R., de Castro M.-J., de Lamas C., Picáns R., Couce M.L. (2020). Effects of prebiotic and probiotic supplementation on lactase deficiency and lactose intolerance: A systematic review of controlled trials. Nutrients.

[87] Oak S.J., Jha R. (2019). The effects of probiotics in lactose intolerance: A systematic review. Crit. Rev. Food Sci. Nutr..

[88] Gingold-Belfer R., Levy S., Layfer O., Pakanaev L., Niv Y., Dickman R., Perets T.T. (2020). Use of a novel probiotic formulation to alleviate lactose intolerance symptoms—A pilot study. Probiotics Antimicrob. Proteins.

[89] He T., Venema K., Priebe M., Welling G., Brummer R.J., Vonk R. (2008). The role of colonic metabolism in lactose intolerance. Eur. J. Clin. Investig..

[90] Di Costanzo M., Canani R.B. (2018). Lactose intolerance: Common misunderstandings. Ann. Nutr. Metab..

[91] D’Auria E., Venter C. (2020). Precision medicine in cow’s milk allergy. Curr. Opin. Allergy Clin. Immunol..

[92] Verduci E., Zuccotti G.V., Peroni D.G. (2022). New Insights in Cow’s Milk and Allergy: Is the Gut Microbiota the Missing Link?. Nutrients.

[93] Koletzko S., Niggemann B., Arató A., Dias J., Heuschkel R., Husby S., Mearin M., Papadopoulou A., Ruemmele F., Staiano A. (2012). Diagnostic approach and management of cow’s-milk protein allergy in infants and children: ESPGHAN GI Committee practical guidelines. J. Pediatr. Gastroenterol. Nutr..

[94] D’Auria E., Salvatore S., Pozzi E., Mantegazza C., Sartorio M.U.A., Pensabene L., Baldassarre M.E., Agosti M., Vandenplas Y., Zuccotti G. (2019). Cow’s milk allergy: Immunomodulation by dietary intervention. Nutrients.

[95] Forsgård R.A. (2019). Lactose digestion in humans: Intestinal lactase appears to be constitutive whereas the colonic microbiome is adaptable. Am. J. Clin. Nutr..

[96] Toca M., Fernández A., Orsi M., Tabacco O., Vinderola G. (2022). Intolerancia a la lactosa: Mitos y verdades. Actualización. Arch. Argent. Pediatría.

[97] Facioni M.S., Raspini B., Pivari F., Dogliotti E., Cena H. (2020). Nutritional management of lactose intolerance: The importance of diet and food labelling. J. Transl. Med..

[98] Singh M., Singh V., Friesen C.A. (2020). Colonic mucosal inflammatory cells in children and adolescents with lactase deficiency. Pathol.-Res. Pract..

[99] Ibrahim S.A., Gyawali R., Awaisheh S.S., Ayivi R.D., Silva R.C., Subedi K., Aljaloud S.O., Siddiqui S.A., Krastanov A. (2021). Fermented foods and probiotics: An approach to lactose intolerance. J. Dairy Res..

[100] Vitellio P., Celano G., Bonfrate L., Gobbetti M., Portincasa P., De Angelis M. (2019). Effects of Bifidobacterium longum and Lactobacillus rhamnosus on gut microbiota in patients with lactose intolerance and persisting functional gastrointestinal symptoms: A randomised, double-blind, cross-over study. Nutrients.

[101] Cancarevic I., Rehman M., Iskander B., Lalani S., Malik B.H. (2020). Is there a correlation between irritable bowel syndrome and lactose intolerance?. Cureus.

[102] Alkalay M.J. (2021). Nutrition in patients with lactose malabsorption, celiac disease, and related disorders. Nutrients.

[103] Jansson-Knodell C.L., White M., Lockett C., Xu H., Shin A. (2022). Associations of food intolerance with irritable bowel syndrome, psychological symptoms, and quality of life. Clin. Gastroenterol. Hepatol..

[104] Usai-Satta P., Lai M., Oppia F. (2022). Lactose malabsorption and presumed related disorders: A review of current evidence. Nutrients.

[105] Efremova I., Maslennikov R., Poluektova E., Vasilieva E., Zharikov Y., Suslov A., Letyagina Y., Kozlov E., Levshina A., Ivashkin V. (2023). Epidemiology of small intestinal bacterial overgrowth. World J. Gastroenterol..

[106] Hodges J.K., Cao S., Cladis D.P., Weaver C.M. (2019). Lactose intolerance and bone health: The challenge of ensuring adequate calcium intake. Nutrients.

[107] Treister-Goltzman Y., Peleg R. (2019). Primary lactase deficiency and bone mineral density in postmenopausal women. Osteoporos. Int..

[108] Ratajczak A.E., Rychter A.M., Zawada A., Dobrowolska A., Krela-Kaźmierczak I. (2021). Lactose intolerance in patients with inflammatory bowel diseases and dietary management in prevention of osteoporosis. Nutrition.

[109] Zingone F., Bertin L., Maniero D., Palo M., Lorenzon G., Barberio B., Ciacci C., Savarino E.V. (2023). Myths and Facts about Food Intolerance: A Narrative Review. Nutrients.

[110] Szilagyi A., Ishayek N. (2018). Lactose intolerance, dairy avoidance, and treatment options. Nutrients.

[111] Malik T.F., Panuganti K.K. (2023). Lactose intolerance. StatPearls [Internet].

[112] Anguita-Ruiz A., Aguilera C.M., Gil Á. (2020). Genetics of lactose intolerance: An updated review and online interactive world maps of phenotype and genotype frequencies. Nutrients.

[113] Wanes D., Husein D.M., Naim H.Y. (2019). Congenital lactase deficiency: Mutations, functional and biochemical implications, and future perspectives. Nutrients.

[114] De Luca P., Iaconis D., Biffali E., Enza C., de Magistris L., Riegler G., Pappalardo D., Amato M.R., Iardino P., Montanino C. (2021). Development of a novel SNP assay to detect lactase persistence associated genetic variants. Mol. Biol. Rep..

[115] Marten L.M., Wanes D., Stellbrinck T., Santer R., Naim H.Y. (2022). Hypomorphic variants of lactase-phlorizin hydrolase in congenital lactase deficiency are trafficking incompetent and functionally inactive. Biochim. Biophys. Acta (BBA)-Mol. Basis Dis..

[116] Hoang T.T., Lei Y., Mitchell L.E., Sharma S.V., Swartz M.D., Waller D.K., Finnell R.H., Benjamin R.H., Browne M.L., Canfield M.A. (2019). Maternal lactase polymorphism (rs4988235) is associated with neural tube defects in offspring in the National Birth Defects Prevention Study. J. Nutr..

[117] Kowalówka M., Kosewski G., Lipiński D., Przysławski J. (2023). A Comprehensive Look at the-13910 C>T LCT Gene Polymorphism as a Molecular Marker for Vitamin D and Calcium Levels in Young Adults in Central and Eastern Europe: A Preliminary Study. Int. J. Mol. Sci..

[118] Domżał-Magrowska D., Kowalski M.K., Małecka-Wojciesko E. (2023). The incidence of adult type hypolactasia in patients with irritable bowel syndrome. Gastroenterol. Rev. Przegląd Gastroenterol..

[119] Comerford K.B., Pasin G. (2017). Gene–dairy food interactions and health outcomes: A review of nutrigenetic studies. Nutrients.

[120] Aruoma O.I., Hausman-Cohen S., Pizano J., Schmidt M.A., Minich D.M., Joffe Y., Brandhorst S., Evans S.J., Brady D.M. (2019). Personalized nutrition: Translating the science of nutrigenomics into practice: Proceedings from the 2018 American College of Nutrition Meeting. J. Am. Coll. Nutr..

[121] Ferguson L.R., De Caterina R., Görman U., Allayee H., Kohlmeier M., Prasad C., Choi M.S., Curi R., De Luis D.A., Gil Á. (2016). Guide and position of the international society of nutrigenetics/nutrigenomics on personalised nutrition: Part 1-fields of precision nutrition. J. Nutr. Nutr..

[122] Robles L., Priefer R. (2020). Lactose intolerance: What your breath can tell you. Diagnostics.

[123] Tomczonek-Moruś J., Wojtasik A., Zeman K., Smolarz B., Bąk-Romaniszyn L. (2019). 13910C>T and 22018G>A LCT gene polymorphisms in diagnosing hypolactasia in children. United Eur. Gastroenterol. J..

[124] Mattar R., de Campos Mazo D.F., Carrilho F.J. (2012). Lactose intolerance: Diagnosis, genetic, and clinical factors. Clin. Exp. Gastroenterol..

[125] Floris M., Cano A., Porru L., Addis R., Cambedda A., Idda M.L., Steri M., Ventura C., Maioli M. (2020). Direct-to-consumer nutrigenetics testing: An overview. Nutrients.

[126] Guasch-Ferré M., Dashti H.S., Merino J. (2018). Nutritional genomics and direct-to-consumer genetic testing: An overview. Adv. Nutr..

[127] Marietta C., McGuire A.L. (2009). Currents in contemporary ethics Direct-to-consumer genetic testing: Is it the practice of medicine?. J. Law Med. Ethics.

[128] De S., Pietilä A.-M., Iso-Touru T., Hopia A., Tahvonen R., Vähäkangas K. (2020). Information provided to consumers about direct-to-consumer nutrigenetic testing. Public Health Genom..

[129] Misselwitz B., Pohl D., Frühauf H., Fried M., Vavricka S.R., Fox M. (2013). Lactose malabsorption and intolerance: Pathogenesis, diagnosis and treatment. United Eur. Gastroenterol. J..

[130] Zheng X., Chu H., Cong Y., Deng Y., Long Y., Zhu Y., Pohl D., Fried M., Dai N., Fox M. (2015). Self-reported lactose intolerance in clinic patients with functional gastrointestinal symptoms: Prevalence, risk factors, and impact on food choices. Neurogastroenterol. Motil..

[131] Tandy-Connor S., Guiltinan J., Krempely K., LaDuca H., Reineke P., Gutierrez S., Gray P., Davis B.T. (2018). False-positive results released by direct-to-consumer genetic tests highlight the importance of clinical confirmation testing for appropriate patient care. Genet. Med..

[132] Kuchay R.A.H. (2020). New insights into the molecular basis of lactase non-persistence/persistence: A brief review. Drug Discov. Ther..

[133] Labrie V., Buske O.J., Oh E., Jeremian R., Ptak C., Gasiūnas G., Maleckas A., Petereit R., Žvirbliene A., Adamonis K. (2016). Lactase nonpersistence is directed by DNA-variation-dependent epigenetic aging. Nat. Struct. Mol. Biol..

[134] Leseva M.N., Grand R.J., Klett H., Boerries M., Busch H., Binder A.M., Michels K.B. (2018). Differences in DNA methylation and functional expression in lactase persistent and non-persistent individuals. Sci. Rep..

[135] Fukushima A., Goda T., Motohashi Y., Sakuma K. (2004). The specific expression patterns of lactase, sucrase and calbindin-D9k in weaning rats are regulated at the transcriptional level. J. Nutr. Sci. Vitaminol..

[136] Motohashi Y., Fukushima A., Kondo T., Sakuma K. (1997). Lactase decline in weaning rats is regulated at the transcriptional level and not caused by termination of milk ingestion. J. Nutr..

[137] Rossi M., Maiuri L., Fusco M.I., Salvati V.M., Fuccio A., Auricchio S., Mantei N., Zecca L., Gloor S.M., Semenza G. (1997). Lactase persistence versus decline in human adults: Multifactorial events are involved in down-regulation after weaning. Gastroenterology.

[138] Xu Y.-J., Wu X. (2015). Foodomics in microbiological investigations. Curr. Opin. Food Sci..

[139] Pimentel G., Burton K.J., Rosikiewicz M., Freiburghaus C., von Ah U., Münger L.H., Pralong F.P., Vionnet N., Greub G., Badertscher R. (2017). Blood lactose after dairy product intake in healthy men. Br. J. Nutr..

[140] Vionnet N., Münger L.H., Freiburghaus C., Burton K.J., Pimentel G., Pralong F.P., Badertscher R., Vergères G. (2019). Assessment of lactase activity in humans by measurement of galactitol and galactonate in serum and urine after milk intake. Am. J. Clin. Nutr..

[141] Bush C.L., Blumberg J.B., El-Sohemy A., Minich D.M., Ordovás J.M., Reed D.G., Behm V.A.Y. (2020). Toward the definition of personalized nutrition: A proposal by the American Nutrition Association. J. Am. Coll. Nutr..

[142] Toro-Martín D., Arsenault B.J., Després J.-P., Vohl M.-C. (2017). Precision nutrition: A review of personalized nutritional approaches for the prevention and management of metabolic syndrome. Nutrients.

[143] Sales N.M.R., Pelegrini P.B., Goersch M. (2014). Nutrigenomics: Definitions and advances of this new science. J. Nutr. Metab..

[144] Ordovas J.M., Ferguson L.R., Tai E.S., Mathers J.C. (2018). Personalised nutrition and health. BMJ.

[145] Authority E.F.S. (2017). Dietary Reference Values for Nutrients Summary Report.

[146] Dekker P.J., Koenders D., Bruins M.J. (2019). Lactose-free dairy products: Market developments, production, nutrition and health benefits. Nutrients.

[147] Fassio F., Facioni M.S., Guagnini F. (2018). Lactose maldigestion, malabsorption, and intolerance: A comprehensive review with a focus on current management and future perspectives. Nutrients.

[148] Suchy F.J., Brannon P.M., Carpenter T.O., Fernandez J.R., Gilsanz V., Gould J.B., Hall K., Hui S.L., Lupton J., Mennella J. (2010). NIH consensus development conference statement: Lactose intolerance and health. NIH Consens. State Sci. Statements.

[149] Nicklas T.A., Qu H., Hughes S.O., He M., Wagner S.E., Foushee H.R., Shewchuk R.M. (2011). Self-perceived lactose intolerance results in lower intakes of calcium and dairy foods and is associated with hypertension and diabetes in adults. Am. J. Clin. Nutr..

[150] Heaney R.P. (2000). Calcium, dairy products and osteoporosis. J. Am. Coll. Nutr..

[151] (2020). Lactose Intolerance—Treatment—NHS. https://www.nhs.uk/conditions/lactose-intolerance/treatment/.

[152] (2020). Guide: Nutritional Characteristics—Parmigiano Reggiano. https://www.parmigianoreggiano.com/product-guide-nutritional-characteristics/#2.

[153] Szilagyi A., Shrier I., Heilpern D., Je J.S., Park S., Chong G., Lalonde C., Cote L.-F., Lee B. (2010). Differential impact of lactose/lactase phenotype on colonic microflora. Can. J. Gastroenterol. Hepatol..

[154] Kok C.R., Hutkins R. (2018). Yogurt and other fermented foods as sources of health-promoting bacteria. Nutr. Rev..

[155] Hertzler S., Savaiano D.A., Dilk A., Jackson K.A., Bhriain S.N., Suarez F.L. (2017). Nutrient considerations in lactose intolerance. Nutr. Prev. Treat. Dis..

[156] Silanikove N., Leitner G., Merin U. (2015). The interrelationships between lactose intolerance and the modern dairy industry: Global perspectives in evolutional and historical backgrounds. Nutrients.

[157] Martini M.C., Lerebours E.C., Lin W.J., Harlander S.K., Berrada N.M., Antoine J.M., Savaiano D.A. (1991). Strains and species of lactic acid bacteria in fermented milks (yogurts): Effect on in vivo lactose digestion. Am. J. Clin. Nutr..

[158] Kies A.K. (2014). Authorised EU health claims related to the management of lactose intolerance: Reduced lactose content, dietary lactase supplements and live yoghurt cultures. Foods, Nutrients and Food Ingredients with Authorised EU Health Claims.

[159] Mutch D.M., Wahli W., Williamson G. (2005). Nutrigenomics and nutrigenetics: The emerging faces of nutrition. FASEB J..

[160] Röttger-Wirtz S., Alie D. (2021). Personalised Nutrition: The EU’s Fragmented Legal Landscape and the Overlooked Implications of EU Food Law. Eur. J. Risk Regul..

[161] Nordström K., Goossens J. (2016). Personalized nutrition and social justice: Ethical considerations within four future scenarios applying the perspective of Nussbaum’s capabilities approach. J. Agric. Environ. Ethics.

[162] Casellas F., Aparici A., Pérez M., Rodríguez P. (2016). Perception of lactose intolerance impairs health-related quality of life. Eur. J. Clin. Nutr..

[163] Enko D., Meinitzer A., Brandmayr W., Halwachs-Baumann G., Schnedl W.J., Kriegshäuser G. (2018). Association between increased plasma levels of homocysteine and depression observed in individuals with primary lactose malabsorption. PLoS ONE.

